# Renal cell carcinoma with early skin metastasis and partial response on tyrosine kinase inhibitor: A case report

**DOI:** 10.1016/j.ijscr.2022.107020

**Published:** 2022-04-05

**Authors:** Agus Rizal Ardy Hariandy Hamid, Reginald Rustandi, Sahat Matondang, Meilania Saraswati, Lenny Sari, Sonar Soni Panigoro

**Affiliations:** a Department of Urology, Faculty of Medicine, Universitas Indonesia, Jakarta, Indonesia; b Department of Radiology, Faculty of Medicine, Universitas Indonesia, Jakarta, Indonesia; c Department of Pathology Anatomy, Faculty of Medicine, Universitas Indonesia, Jakarta, Indonesia; d Department of Anatomic Pathology, Pondok Indah Hospital, Jakarta, Indonesia; e Department of Surgery, Oncology Division, Faculty of Medicine, Universitas Indonesia, Jakarta, Indonesia

**Keywords:** Advanced cancer, Urology, Renal cell carcinoma, Chemotherapy, Skin metastasis, Oncology

## Abstract

**Introduction and importance:**

Renal cell carcinoma (RCC) skin metastasis is a rare disease. However, there are no data on the effect of a Tyrosine Kinase Inhibitor (TKI) on its treatment.

**Case presentation:**

A 54-year-old male patient with renal cell carcinoma developed subcutaneous metastasis three months after radical nephrectomy and there was no discoloration or pain. Furthermore, an excision biopsy confirmed the metastatic lesion, and pazopanib was initiated as a treatment method. After 1-month of treatment, the patient developed ulceration and subsided after treatment was stopped. Similarly, a follow-up PET scan was performed almost a year after stopping the treatment, which showed improvement over metastatic pulmonary lesions.

**Clinical discussion:**

Renal cell carcinoma (RCC) major metastases were observed in pulmonary, costal, and skin. Tumor burden and location of metastasis influences progression free-survival of RCC patients treated with TKI.

**Conclusion:**

In this case, TKI treatment showed a long-term partial response, despite its lack of continuous therapy.

## Highlights

• Skin metastases developed after left radical nephrectomy with clean surgical margin
• Three different metastatic sites were found with different prognostic rate
• Tyrosine kinase inhibitor showed partial response over lung and skin metastases

## 1 Introduction

Clear cell renal cell carcinoma (RCC) is a malignant disease that is highly resistant to chemotherapy, which can spread to various organs such as abdominal lymph nodes, liver, adrenal glands, pancreas, spleen, peritoneum, lungs, pleura, brain, bone, thyroid, and even skin tissue [2]. No current data has shown the effect of skin metastases on the tyrosine kinase inhibitor (TKI) treatment. Therefore, the study aims to report the long-term partial response of early skin metastases to nephrectomy of the organ-confined tumor mass, which showed partial response to the TKI.

## 2 Method

This work has been reported in line with the SCARE 2020 criteria [1].

## 3 Case presentation

**Fig. 1:**
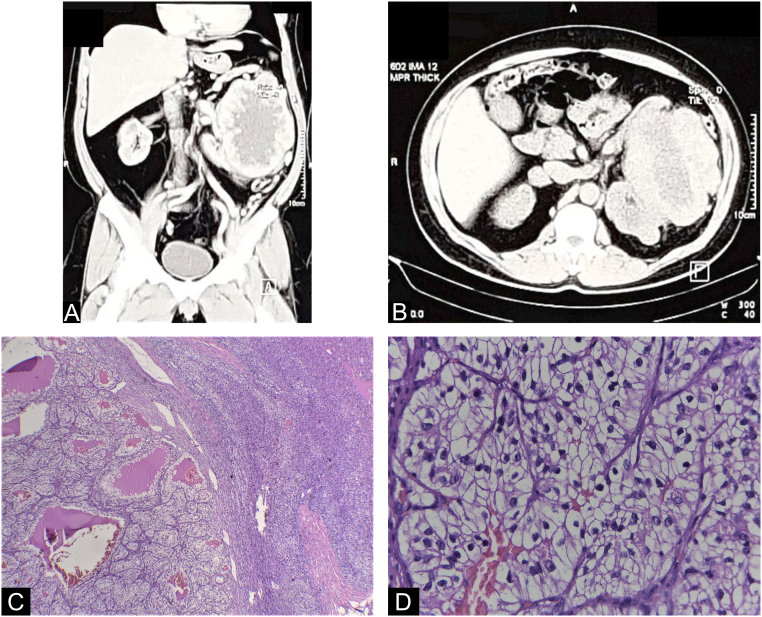
(A) Left Renal Tumor on Coronal Plane CT-Scan with a size of 11.3 × 14.4 × 8.8 cm with contrast enhancement, no adrenal tissue could be identified suggestive that the tumor infiltrates the adrenal tissue. (B) Left Renal Tumor on Axial Planet CT-Scan, showing an inhomogen hipodense tumor occupied mostly on lateral side of the left kidney, expanding over the fascia gerota. (C) Confirmation of histological evaluation of left renal tumor infiltrates the adrenal tissue. (D) 40× magnification of the left renal tumor showed prominent nucleoli. Therefore, it was graded as WHO/ISUP grade 2.

**Fig. 2:**
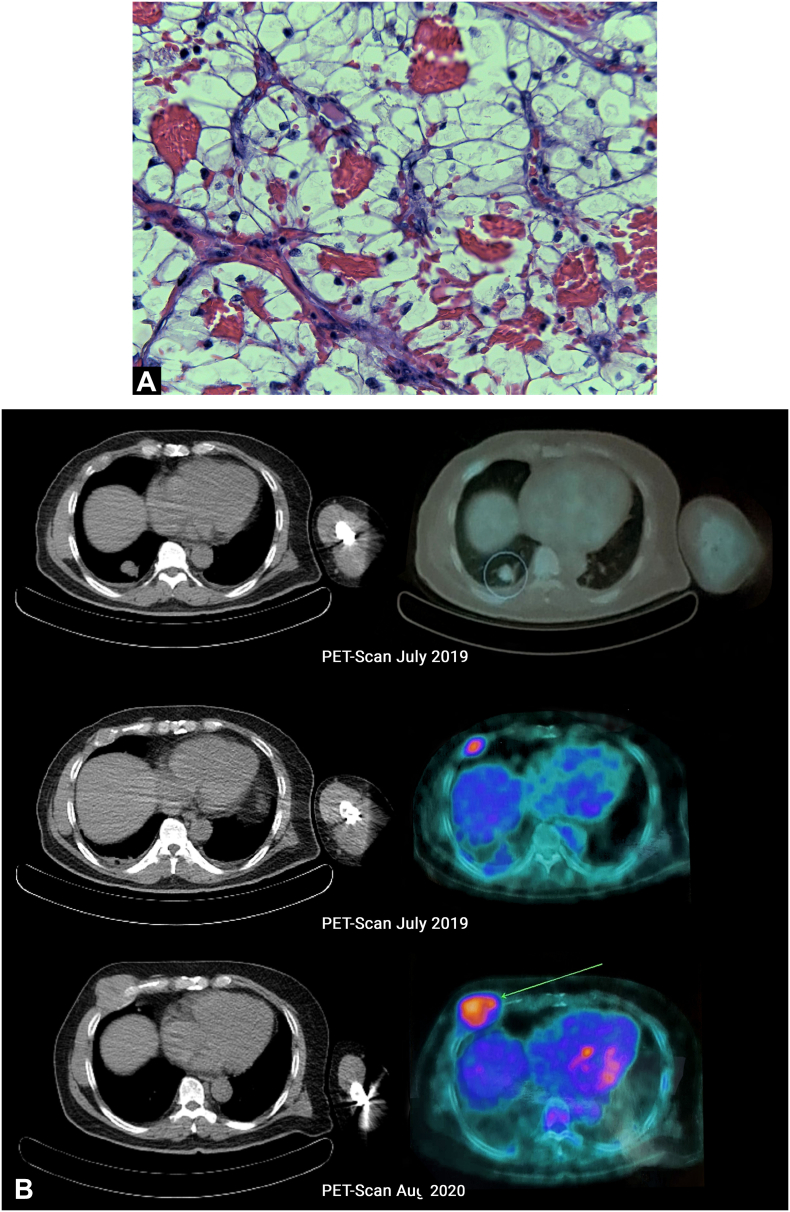

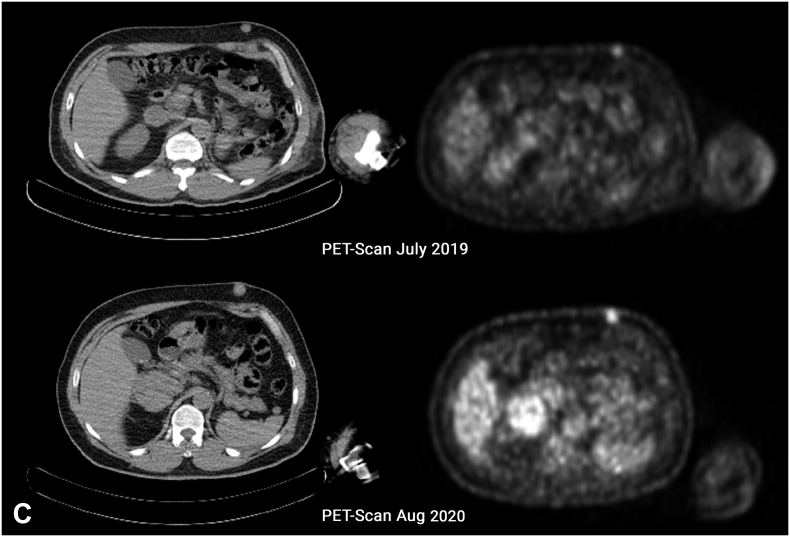
(A) 40× magnification of left chest tumor showed prominent nucleoli and same characteristic that consist of solid cells with thin fibrovascular septa compared to the 40× magnification of the left renal tumor. (B) PET-Scan (July 2019) shows metabolic active multiple nodules on the 9th and 10th segment of the right lung and an active metabolic mass with a diameter of 2,6 cm on the 5th right anterior costae; On follow-up, PET-scan (August 2020) showed complete resolution of multiple nodules on the 9th and 10th segment of the right lung and progression of mass on the 5th right anterior costae to 5,7 cm (C) a mobile, firm, with a diameter of 1,3 cm metabolic active skin nodule is seen on the left upper abdominal and on follow-up PET-Scan its size increase to 1,8 cm.

**Fig. 3:**
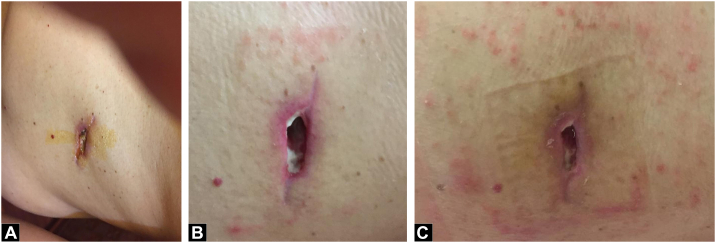
(A) 1 month after ingestion of Pazopanib as systemic therapy, the patient began to develop ulceration over the surgical biopsy site on his left abdomen. (B) Four days after discontinuation of Pazopanib, wound improvement was shown. (C) Ulceration was almost completely resolved after eight days of Pazopanib discontinuation.

A 54-year-old former male smoker was diagnosed with Clear Cell RCC, cT4N0M0 Fig. 1(A) & (B), and radical left nephrectomy was performed in February 2019. The histopathological result showed a WHO/ISUP grade II pT4 with infiltration into the adrenal gland. Fig. 1(C) & (D) Infiltration into other adjacent structures was not shown with clear surgical margins, and no regional lymph nodes were involved. Furthermore, after three months (May 2020), based on the histopathological result, a metastatic lesion of Clear Cell RCC was reported as a skin nodule on his left chest and abdomen Fig. 2(A). Systemic therapy was first declined, and the patient gave informed consent for this publication for educational purposes. Furthermore, after three months, PET-scan confirmed multiple nodules on the 9th and 10th segment of the right lung Fig. 2(B) a mass on 5th anterior right costae Fig. 2(B) and a skin nodule on left upper abdomen Fig. 2(C) then the patient agreed to take 2 × 400 mg pazopanib. Ulceration develops unexpectedly after one month during the skin excisional biopsy Fig. 3(A). Erythema surrounding the edge of the scar was observed first, then exudative ulceration appeared. In addition, the patient was instructed to wash the wound twice daily with saline solution, povidone‑iodine, and then cover the wound with a waterproof bandage. Systemic treatment with pazopanib was postponed, and four days after, the ulceration began to shrink. No pus was reported Fig. 3(B). On day 8th, the wound diameter was reduced, with no erythema, and the wound was dry Fig. 3(C).

One year after stopping the systemic therapy, a mass was observed on his 5th anterior right ribs. A second PET scan was performed, multiple nodules on the 9th, 10th segment of the right lung completely disappeared Fig. 2(B). Both mass on the 5th anterior right costae Fig. 2(B) and a skin nodule on the upper left abdomen increased in size Fig. 2(C). On this basis, the patient was offered and agreed to continue 1 × 400 mg pazopanib. Furthermore, a monthly check-up was scheduled, a decrease in mass on the anterior right costae was observed by the patient after a month of TKI treatment. However, patient choose to stop the medication.

## 4 Discussion

This case report showed that three major metastases were observed after radical nephrectomy: pulmonary, costal, and skin. Furthermore, this may be due to Circulating Tumor Cells (CTC), malignant cells in the peripheral blood that originate from primary tumours or metastatic sites [2]. Most of these CTC are destroyed in the circulation through attack by T cells, natural killer cells, shear forces, and oxidative stress [2]. In contrast, others can develop abilities to survive through epithelial-to-mesenchymal transition (EMT) [2] and protective factors provided by neutrophil extracellular traps (NETs) [2]. These two pathways act as camouflage to ensure survivability to avoid immune responses. The CTC can explain the spread over the various metastatic sites. Recent studies have shown three possible methods to differentiate between epithelial markers, genetic modification by RT-PCR and a combination of morphological and genetic analysis [3]. Due to the elusive impact of the RCC in nature, none of these approaches can be precise. The identification of the epithelial cell adhesion molecule/EpCAM is difficult due to the behavior of RCC to pass through EMT, resulting in the loss of its epithelial antigen. Despite their inability to differentiate benign kidney disease, other alternative markers such as carbonic anhydrase 9 (CAIX) or CD147 showed >97% expression in clear cell RCC samples [4]. The development of CTC analysis can benefit diagnostic and treatment tools. However, further research is needed for the application of CTC in the clinical environment.

A recent prospective study has shown that tumor burden and location of metastasis influence the rate of progression-free survival of patients with metastatic RCC using tyrosine kinase inhibitor (TKI), with pulmonary metastasis as the only positive predictor and skin as the worst predictor [5], [6]. Although due to the limited investigation of skin metastasis, this case report shows regression of skin metastasis after the administration of pazopanib. However, pazopanib has been used infrequently; it was only consumed for a month (2 × 400 mg). Furthermore, it was later stopped due to the growth of ulceration at the post tumor excision in the skin and there is no new lesion on this site. After stopping for a year, a regression in some sites of metastasis and a progression in other sites were reported. Similarly, a new lump was reported under the right hemiabdomen's skin. An interesting result showed that this lump has improved after continuing pazopanib treatment. This case report may shed new light on pazopanib (TKI) treatment in case of skin metastasis, despite its lack of continuous consumption.

## 5 Conclusion

A long-term partial response of early skin metastasis in RCC using TKI was reported, despite its lack of continuous treatment.

## Sources of funding

This work was supported by 10.13039/501100006378Universitas Indonesia for funding this research through PUTI Grant with contract number NKB-2281/UN2.RST/HKP.05.00/2020.

## Ethical approval

The Ethics Committee of the Faculty of Medicine, Universitas Indonesia, approved the study protocol (KET-144/UN2.F1/ETIK/PPM.00.02/2020).

## Consent

This report has received written consent from the patient to publish case details and any accompanying images published. The author ensures the confidentiality of the patient's identity in this report.

## Author contribution

Agus Rizal Ardy Hariandy Hamid: Conceptualization, Methodology, Validation, Resources, Supervision.

Reginald Rustandi: Methodology, Writing-Review and editing, Resources.

Sahat Matondang: Writing-Review and editing, Resources.

Meilania Saraswati: Writing-Review and editing, Resources.

Lenny Sari: Writing-Review and editing, Resources.

Sonar Sonny Panigoro: Writing Original-Draft, Investigation.

## Research registration

Not applicable.

## Guarantor

Agus Rizal Ardy Hariandy Hamid.

## Provenance and peer review

Not commissioned, externally peer-reviewed.

## Declaration of competing interest

The authors had reported no conflicts of interest in this work.
